# A cohort study of quality of life in partners of young breast cancer survivors compared to partners of healthy controls

**DOI:** 10.1186/s41687-020-0184-4

**Published:** 2020-03-06

**Authors:** Andrea Cohee, Susan Storey, Joseph G. Winger, David Cella, Timothy Stump, Patrick O. Monahan, Victoria L. Champion

**Affiliations:** 1 Indiana University School of Nursing, 600 Barnhill Drive, Indianapolis, IN 46202 USA; 2 Department of Psychiatry and Behavioral Sciences, Duke University Medical Center, Durham, North Carolina USA; 3 Department of Medical Social Sciences, Northwestern University, Chicago, Illinois USA; 4 Department of Biostatistics, Indiana University School of Medicine, Indianapolis, Indiana USA

**Keywords:** Breast cancer, Partners, Acquaintance control, Quality of life

## Abstract

**Background:**

Partners of young breast cancer survivors (BCS) are at increased risk for deficits in quality of life (QoL). To intervene effectively, it is important to understand how the breast cancer experience impacts partners. The purpose of this study was to compare QoL between partners of young BCS and partners of healthy acquaintance controls.

**Methods:**

Partners of young BCS (3–8 years post treatment and ≤ 45 years old at diagnosis) and partners of age-matched healthy acquaintance controls completed questionnaires on overall, physical (physical function, sexual difficulty), social (personal resources, sexual enjoyment, marital satisfaction, partner social support, social constraints, parenting satisfaction), psychological (depressive symptoms), and spiritual (behaviors, beliefs, and activities) QoL. Analyses included descriptive statistics and one-way ANOVA to compare partner groups on all study variables.

**Results:**

Although partners of young BCS (*n* = 227) reported fewer social constraints (*p* < .001), they reported lower overall QoL (p < .001), fewer personal resources (p < .001), more sexual difficulty (*p* = .019), less sexual enjoyment (*p* = .002), less marital satisfaction (p = .019), more depressive symptoms (*p* = .024), and fewer spiritual behaviors (*p* < .001), beliefs (*p* = .001) and activities (*p* = .003) compared to partners of healthy acquaintance controls (*n* = 170). Additional analysis showed that perceptions that the relationship changed for the better since cancer, social constraints, partner social support, and depression predicted marital satisfaction among partners of young BCS.

**Conclusions:**

Partners of young BCS are at risk for poorer overall, physical, social, psychological, and spiritual QoL compared to partners of healthy women. Interventions targeting QoL domains may enable partners to effectively support their partner and improve their QoL.

## Introduction

Approximately 3.4 million women in the United States are breast cancer survivors (BCS) [1]. About 89% of all women diagnosed can expect to survive more than 5 years post-treatment [2]. Although women are surviving longer, BCS are at risk for poor physical, social, psychological, and spiritual quality of life (QoL) years after the cancer experience [3].

Many BCS have partners who also experience deficits in QoL years after the cancer experience [4]. Although limited, research has demonstrated that partners of BCS diagnosed before age 50 are disproportionately affected by cancer, as they report more QoL deficits than partners of BCS diagnosed at a later age [4]. Longitudinal studies have found that partners of young BCS have worse marital functioning, more posttraumatic stress hyperarousal, and worse overall QoL than partners of older BCS [5]. However, predictors of QOL are likely to vary greatly between partners of younger BCS and partners of older BCS, given that life circumstances and demands vary. Understanding ways in which the breast cancer experience affects young survivors’ partners’ QoL requires comparisons between partners of BCS and partners of healthy women. To date, the literature comparing partners of young BCS to partners of age-matched controls is lacking.

The aim of the current study was to compare partners of young BCS to partners of healthy acquaintance controls on long-term QoL (overall, physical, social, psychological, and spiritual). The knowledge gained from this study will help clinicians who strive to meet the needs of younger BCS and their partners.

### Theoretical framework

The City of Hope Quality of Life Model, which guided the parent study [3], posits that four domains of well-being contribute to a person’s overall QoL [6, 7] [3, 4, 8]. The four domains are physical wellbeing (maintaining function and independence), social wellbeing (relationships with others), psychological wellbeing (mental health), and spiritual wellbeing (existential and religious dimensions) [7]. Together, these domains predict overall QoL.

## Methods

### Participants

This project was part of a larger study [1] examining QoL among BCS diagnosed 3–8 years prior. The purpose of the parent study was to compare long-term QoL in younger BCS (women diagnosed with breast cancer at age 45 years or younger) to older BCS (women diagnosed with breast cancer at ages 55–70) and age-matched healthy acquaintance controls (women within 5 years of survivors’ age who had not been diagnosed with breast cancer). Briefly, Eastern Cooperative Oncology Group-American College of Radiology Imaging Network (ECOG-ACRIN) generated a list of eligible BCS to whom a study brochure was mailed. One week after the mailing, research assistants called BCS to determine interest. If BCS expressed interest, research assistants mailed informed consent to BCS, along with a postage-paid return envelope. Once signed consents were received, questionnaires were mailed to BCS. Age-matched healthy acquaintance controls were nominated by enrolled BCS. All other procedures for consenting and collecting data from healthy acquaintance controls were the same. Data were collected 2004–2010. Additional details about the parent study are reported elsewhere [3].

Analyses for the current study focused on partners of young BCS and partners of healthy acquaintance controls. A list of young BCS and healthy acquaintance controls, who reported that they lived with a partner or spouse, was extracted from the larger dataset. Using the same recruitment procedures, research assistants invited partners to participate in the study. The partners of young BCS and age-matched healthy acquaintance controls did not have to be of the same age. Rather, partners had to verify that they were married or in a serious committed relationship with the survivor or healthy acquaintance control. If partners expressed interest and consented, research assistants mailed partners questionnaires to complete. Of the 417 married/partnered young breast cancer survivors, 227 had partners who were willing to participate (54.4% response rate). Of the 324 healthy acquaintance controls who were married/partnered, 170 had partners who participated (52.4% response rate).

### Measures

Socio-demographic information, including current age, education, race, ethnicity, and religious affiliation, was collected from partners of young BCS and partners of healthy acquaintance controls. Partners of young BCS were also asked if their relationship since cancer had changed for the better, gotten worse, or stayed the same. Gender was not recorded for either partner group. The City of Hope Quality of Life Model [6, 7], provided structure for assessing each QoL domain. QoL domains were measured with the scales described below. Note, for scales that asked partners of young BCS to reflect on the breast cancer experience, partners of healthy controls were asked to reflect on a “stressful event in the last 5 years.” If completed in order, partners could complete the entire questionnaire within 45 min. Measures were grouped and ordered by physical, psychological, social, spiritual, and overall QoL. Partners were encouraged to take breaks between each QoL section.

#### Overall quality of life

Overall QoL was measured by the *Index of Well-Being* (IWB) [9]. The 7-item measure asked how the participant felt about his/her life in general. Mean values were calculated with higher scores indicating better QoL (α = 0.92).

#### Physical QoL

Two aspects of physical functioning were assessed. First, general physical functioning was measured with the 10-item Medical Outcomes Study *Physical Functioning Scale* (PF-10) [10]. The scores are summed and scored positively (α = .92) [10]. Second, sexual difficulty, the physical component of sexual functioning, was assessed with the 3-item *Sexual Difficulty* subscale, a measure adapted from a previously validated scale [11] for use in the parent study [3]. The questions asked about the participant’s difficulty becoming aroused and having an orgasm (α = .85). The three items were summed with a higher score indicating more difficulty.

#### Social QoL

This domain consisted of personal resources, sexual enjoyment, marital satisfaction, partner social support, social constraints, and parenting satisfaction. The 15-item *Personal Resource Questionnaire* measured support from other people such as friends, co-workers, relatives, and others in the participant’s social network [12]. The sum of the 15 items was taken with a higher score indicating greater support from personal resources (α = .91). Sexual enjoyment, the social component of sexual functioning, was measured with a modified 4-item *Sexual Enjoyment* subscale adapted from a previously validated measure [11]. The subscale asked participants about interest in sexual activities, ability to relax and enjoy sexual activities, satisfaction with frequency of sexual activities, and frequency of sexual thoughts or fantasies. This checklist was summed with higher scores relating to more enjoyment and satisfaction.

Marital satisfaction was measured with the previously validated 15-item *Evaluating and Nurturing Relationship Issues, Communication, and Happiness Marital Satisfaction Scale (ENRICH MSS).* The items were summed with higher scores indicating greater marital satisfaction (α = .91) [13]. Perceived social support from the partner was measured with the 7-item *Northouse Social Support Scale,* which was developed and validated in cancer populations [14]. Higher scores indicated greater social support (α = .84). Social constraints were measured using 14 items from the *Lepore Social Constraints Scale,* which has been validated in prior oncology populations [15]. The scale asked participants how often they perceived behaviors that limited discussions of cancer or a serious life event (i.e. avoidance, denial, problem minimization) from their partner in the last four weeks (α = .89). Scores were the sum of all items with higher scores indicating more social constraints. The *Parenting Satisfaction Questionnaire asked* participants to describe satisfaction with their parenting abilities. The sum of the 5 items was calculated with higher scores indicating more parenting satisfaction (α = .84).

#### Psychological QoL

This domain was measured using the *Centers for Epidemiologic Studies-Depression Scale* [16], a well-used, 20-item summated scale of depressive symptoms. Scores above 16 were consistent with clinical depression (α = .87).

#### Spiritual QoL

The *Reed Spiritual Perspectives Scale,* previously developed and validated, was composed of 10 items, 4 describing spiritual behaviors (prayer, spiritual discussions with family and friends) and 6 describing spiritual beliefs [17]. Mean values were calculated, with responses ranging 1–6 for each item. Two items were added to describe involvement in spiritual activities such as attending religious events (α = .96).

### Statistical analysis

All analyses were conducted using SPSS 25 [18]. Descriptive statistics, including frequencies and measures of central tendency, were computed for all demographic variables, including age, race, ethnicity, years of education, and religious affiliation. Analysis of variance was used to test group differences in QoL between partners of young BCS and partners of healthy acquaintance controls, with a Bonferroni post hoc analysis (which adjusts for multiple comparisons) was used to identify specific differences between groups.

## Results

### Sample characteristics

The majority of partners of BCS (*n* = 227) and partners of healthy acquaintance controls (*n* = 170) were well educated (mean = 15 years, SD = 2.7), white (92%), and Christian (84%), with an average age of 48 years (SD = 7.8). On average, partners of BCS were 5.5 years out from their partner’s breast cancer diagnosis. There were no significant group differences on demographic variables. Table 1 provides a full list of demographics and group differences.

**Table 1:** Demographic information for partners of young breast cancer survivors and partners of healthy acquaintance controls

Variable	YP (*n* = 227)	AP (*n* = 170)	T-test (*p*) between YP and AP
Race, Number (percentage)			0.404
Caucasian	210 (92.5)	155 (91.2)	
Black or African American	7 (3.1)	6 (3.5)	
Asian	2 (.9)	1 (0.6)	
Other	8 (3.5)	8 (4.7)	
Education (years), mean (*SD*)	14.88 (2.552)	15.32 (2.85)	0.113
Highest Level of Education Completed	N (%) YP	N (%) OP	
Graduate or Professional Degree	38 (16.7)	39 (22.9)	
Some Graduate School	10 (4.4)	5 (2.9)	
Bachelors Degree	58 (25.6)	50 (29.4)	
Associates Degree	21 (9.3)	10 (5.9)	
Some College	31 (13.7)	27 (15.9)	
Technical or Trade School	23 (10.1)	9 (5.3)	
High School Graduate/GED	40 (17.6)	25 (14.7)	
Some High School	5 (2.2)	4 (2.4)	
Elementary School or Less	0	1 (0.6)	
Missing	1 (.4)	0	
Religious Affiliation, Number (percentage)			0.225
Christian	189 (83.6)	144 (84.7)	
Other	11 (9.8)	5 (2.94)	
No religious affiliation	26 (11.5)	21 (12.35)	
Missing	*1 (.44)*		
Current age years, mean (*SD*)	48.04 (7.181)	47.36 (8.625)	0.392

### QoL differences between groups

Because groups were not significantly different on any demographic variable, no variables were entered into analyses as covariates. Table 2 reports the mean differences and associated Cohen’s d effect sizes between partner groups for all scales. Compared to their counterparts, partners of BCS generally reported worse functioning. In each QoL domain, significant differences between partners of young BCS and partners of healthy acquaintance controls were found. All significant group differences were small in effect size using the standard for reporting Cohen’s d.

**Table 2:** Differences between Partners of Young Breast Cancer Survivors and Partners of Healthy Acquaintance Controls

Measure	Mean (*SD*), Rang Partners of young BCS	Mean (*SD*), Range Partners of Healthy Acquaintance Controls	ANOVA^a^ (*p*) between Partner Groups	Cohen’s d
Physical				
Physical Functioning Scale- 10	28.117 (3.498), 11–30	28.45 (3.15), 12–30	−.987 (.324)	na
Sexual Functioning Difficulty	5.652 (3.033), 3–15	4.98 (2.54), 3–14	**2.41 (.016)**	0.24
Social				
Sexual Functioning Total	26.642 (5.073), 9–35	28.21 (4.34), 13–35	**−3.3 (.001)**	−0.17
Sexual Functioning Enjoyment	14.297 (2.818), 6–20	15.18 (2.75), 8–20	**−3.13 (.002)**	−0.32
ENRICH Marital Satisfaction	51.107 (12.941), 11–87.02	54.12 (12.14), 13.3–84.4	**−2.36 (.019)**	− 0.24
Northouse Social Support Scale	26.608 (5.241), 8–35	27.33 (4.75), 14–35	−1.41 (.160)	na
Lepore Social Constraints Scale	20.333 (6.336), 14–40	23.38 (7.86), 14–49	**−4.14 (<.001)**	−0.43
Parenting Satisfaction	20.11 (3.66), 7–25	20.58 (3.33), 10–25	−1.24 (.216)	na
Personal Resource Questionnaire	79.222 (14.809), 20–105	85.13 (11.51), 47–105	**−4.47 (<.001)**	−0.45
Psychological				
Centers for Epidemiologic Studies- Depression Scale	8.795 (8.486), 0–42	7.01 (6.74), 0–33	**2.34 (.020)**	0.23
Spiritual				
Reed Spiritual Perspectives Scale	3.861 (1.396), 1–6	4.37 (1.39), 1–6	**−3.6 (<.001)**	−0.18
Beliefs	4.023 (1.466), 1–6	4.54 (1.37), 1–6	**−3.53 (<.001)**	−0.36
Activities	2.874 (1.423), 1–6	3.32 (1.49), 1–6	**−3.03 (.003)**	−0.31
Behaviors	3.613 (1.462), 1–6	4.12 (1.55), 1–6	**−3.32 (.001)**	−0.34
Overall Wellbeing				
Index of Wellbeing	10.747 (2.359), 3.73–14.70	11.62 (1.91), 6.8–14.7	**−4.08 (<.001)**	−0.41

*Overall well-being.* Compared to partners of healthy acquaintance controls, partners of young BCS reported lower overall QoL (*p* < .001). *Physical well-being*. No group differences were found on general physical functioning. However, partners of young BCS had more sexual difficulty (*p* = .019). *Social well-being.* Partners of young BCS had less sexual enjoyment (*p* = .002), fewer personal resources (*p* < .001), lower marital satisfaction (p = .019), and fewer social constraints (p < .001) compared to partners of healthy acquaintance controls. No other significant differences in social well-being were found. *Psychological well-being.* Compared to partners of healthy acquaintance controls, partners of young BCS reported poorer psychological well-being, as evidenced by more depressive symptoms (*p* = .024). *Spiritual well-being*. Finally, partners of young BCS reported fewer spiritual behaviors (*p* < .001), beliefs (*p* = .001), and activities (*p* = .003).

### Marital satisfaction

Because there were group differences on marital satisfaction and other relationship-focused variables, we conducted additional analyses to explore predictors of the marital relationship. Analyzing partners of young BCS and partners of healthy controls separately, we regressed marital satisfaction on all demographic (age, education, race, ethnicity, religious affiliation), physical (physical functioning and sexual functioning difficulty), psychological (depressive symptoms), spiritual (beliefs, behaviors, and activities), and social (sexual functioning enjoyment (social support and social coonstraints). Variables were removed from the model using a backward selection method until the final models were reached.

For partners of young BCS, marital satisfaction was predicted (r^2^ = .32 F = 24.06 [*p* < .001]) by the partner responding that the marital relationship changed for the better since cancer (β = −.15; *p* = .018), lower depression scores (β = −.25, *p* < .001), fewer social constraints (β = −.16, *p* = .02), and greater social support from the survivor (β = .24, *p* < .001). The regression analysis is presented in Table 3.

**Table 3:** Regression Analysis for Marital Satisfaction in Partners of Young Breast Cancer Survivors

	*B*	*SE B*	β
Social Support	.57	.17	.24^a^
Depression	−.38	.10	-.25^a^
Relationship Changed for the Better	−2.8	1.17	-.15^b^
Social Constraints	−.33	.14	-.16^b^

R^2^ = .32 (*p*s < .05); F(4, 211) = 24.06; *p* < .001

^a^*p* < .001

^b^*p* < .05

For partners of healthy controls, marital satisfaction was predicted (r^2^ = .28 F = 32 [*p* < .001]) by low sexual functioning difficulty (β = −.22, p < .001), and greater social support from the partner (β = .47, p < .001). The regression analysis is presented in Table 4.

**Table 4:** Regression Analysis for Marital Satisfaction in Partners of Healthy Acquaintance Controls

	*B*	*SE B*	β
Social Support	1.21	.17	.47^a^
Sexual Functioning Difficulty	−1.05	.32	-.22^a^

R^2^ = .28 (*p*s < .05); F(2, 166) = 32; *p* < .001

^*a*^*p* < .001

^*b*^*p* < .05

## Discussion

This study was the first to explore QoL (physical, psychological, spiritual, social, and overall) differences between partners of young, long-term breast cancer survivors and partners of healthy acquaintance controls. Partners of young survivors reported worse functioning in at least some aspect of every QoL domain. Partners of young breast cancer survivors reported worse marital satisfaction than partners of healthy controls. Furthermore, differences in the marital relationship and what contributed to marital satisfaction for each group were explored.

### Overall QoL

This study was the first to explore QoL (overall, physical, social, psychological, and spiritual) differences between partners of young, long-term BCS and partners of healthy acquaintance controls. We found that partners of young BCS reported worse functioning in at least some aspect of each QoL domain. Additionally, partners of young BCS reported worse marital satisfaction than partners of healthy controls. Among partners of young BCS, social support from the survivor, social constraints, perceptions that the relationship changed for the better since cancer, and depression contributed to marital satisfaction.

### Overall QoL

Partners of young BCS reported worse overall QoL than partners of healthy acquaintance controls. QoL is, by definition, associated with optimal functioning in all domains [6, 7, 19]. Given the lower QoL scores in each domain, it is not surprising that partners of BCS also reported worse overall QoL.

### Physical QoL

While partners of young BCS reported more sexual difficulty, no other physical functioning differences were reported, which is in line with previous work [20]. Only one other study examined physical functioning in partners at one year post-diagnosis. Shor and colleagues found lower overall physical functioning among BCS partners compared to healthy controls [21]. In that study, many of the BCS were still in active treatment, a very stressful time for BCS and partners alike, which may have contributed to their poorer physical functioning. With regards to sexual difficulty, previous studies have identified erectile dysfunction in up to two-thirds of partners of BCS [22]. Sexual problems have been associated with depressive symptoms, which many partners of young BCS commonly report [23].

### Psychological QoL

Partners of young BCS reported more depressive symptoms than partners of healthy acquaintance controls [24]. Other studies have shown that nearly a third of partners expressed depressive symptoms within the first year after the survivor’s breast cancer diagnosis [19, 20]. Many studies across cultures also have reported higher depressive symptoms in partners of BCS compared to partners of healthy women [21, 25, 26] and similar levels of depressive sympmtoms as the BCS themselves [27].

### Social QoL

Partners of young BCS reported less sexual enjoyment, less interest in sexual activities, less satisfaction with the frequency of sexual activity, and less frequently having sexual thoughts or fantasies than partners of healthy controls. Lower sexual satisfaction among partners has been associated with lower overall sexual functioning, arousal, and sexual satisfaction among BCS [22].

Partners of young BCS expressed having fewer personal resources, or less support in their social network, which is common among partners and caregivers during and immediately following cancer treatment. This perceived lack of a supportive network is attributed to diminished social, leisure, and relaxation activities because of caregiving demands [28]. Results of the present study extend the current literature by showing this isolation may persist years after treatment.

Finally, partners of young BCS scored lower for marital satisfaction than AP. Because there were group differences in marital satisfaction and relationship-focused variables, we explored group differences in the marital relationship further.

### Spiritual QoL

Partners of young BCS reported fewer spiritual behaviors, spiritual beliefs, and spiritual activities. These findings are consistent with previous studies that suggested spiritual needs are common among caregivers of patients with cancer [5, 29]. Caring for a loved one with cancer can impact how caregivers view themselves, others, and the world, at times resulting in spiritual and existential distress (e.g., a loss of meaning and purpose in life) [30, 31]. Low levels of spiritual well-being in cancer caregivers have been associated with a range of negative mental health outcomes [32–34]. It is important to note that these previous studies primarily examined caregivers of patients actively in treatment and/or close to their diagnosis. Our current findings suggest spiritual concerns may persist for many years. More research is needed to replicate and futher characterize these findings.

### Marital satisfaction

Notably, partners of young BCS reported worse marital satisfaction than partners of healthy acquaintance controls. However, in the parent study, the survivors and healthy acquaintance controls to whom these partners were matched did not differ on marital satisfaction [3].

Marital satisfaction for both partners of young BCS and partners of healthy acquaintance controls was predicted by social support from the spouse/partner. For partners of healthy of acquaintance controls, less sexual functioning difficulty uniquely predicted marital satisfaction. For partners of young BCS, additional unique factors including both personal (e.g., depression) and interpersonal factors (e.g., relationship changed for the better since cancer and fewer social constraints) contributed to marital satisfaction. Our results complement previous work. In one study, 74.6% of partners reported that cancer had changed their relationship with 8.6% indicating the relationship had only changed for the worse [35]. In another study, Segrin and colleagues found that partners of women recently diagnosed with breast cancer, relationship satisfaction was associated with better mental health [36]; specifically, low depression scores in partners of young BCS were associated with higher marital satisfaction. However, that team also found that distress levels subsided over time [36]. Partners in the current study were an average of 5.5 years out from their spouse/partner’s breast cancer diagnosis, and many still reported clinically significant depression [4] and marital dissatisfaction.

### Strengths and limitations

Despite the many strengths associated with this large data set, there were also limitations. First, data were cross-sectional from a non-experimental cohort study, and thus this limits the ability to determine causal relationships between cancer experience and QoL. Second, because the data were cross-sectional 3–8 years after the cancer survivor’s diagnosis, any potential differences in partner groups prior to the diagnosis could not be captured. This may be why effect sizes were small between groups. Third, partner groups and their female partners [3] were primarily Caucasian and more highly educated than the average American, which may make them less representative of the general population. Fourth, income, which may contribute to QoL, was not requested from partners. Finally, partners of healthy acquaintance controls were asked to reflect on a “stressful life event” for several of the scales, including social constraints and social support scales, where partners of young long-term BCS were asked to reflect specifically on the breast cancer diagnosis. Without knowing the type of event partners of healthy acquaintance controls used for their reflection, it is difficult to compare the severity of experiences.

## Conclusions

Partners of young BCS and partners of healthy acquaintance controls differed greatly, with partners of young BCS fairing worse in overall, physical (sexual difficulty), social (personal resources, sexual enjoyment, marital satisfaction, and social constraints), psychological (depression), and spiritual (behaviors, beliefs, and activities) QoL. Additionally, marital satisfaction for partners of young BCS was predicted by both personal and interpersonal factors. Notably, these QoL deficits were observed even though survivors were an average of 5.5 years out from diagnosis, which speaks to the long-term effects of cancer on partners.

Healthcare providers should be aware of these effects and include partners in their assessment of QoL deficits, including marital satisfaction. Further research is needed on intervention strategies for partners of BCS. Specifically, tailoring programs to meet partner needs may be especially beneficial [37], as partners express diverse needs [38, 39] that differ during treatment (i.e. finding parking, making appointments, and dealing with insurance questions) and following treatment (i.e. handling fear of recurrence and depression) [40]. Three approaches may be useful for supporting partners of BCS.

First, psychoeducational support is one of the most widely used therapeutic approaches for partners of cancer survivors [41]. These interventions usually combine education about cancer treatment and symptoms [42], the lasting effects of cancer on the partner, social support, intimacy [43], communication, sexuality [44] and more [45]. Studies of psychoeducational support have shown a small to medium effect size [46]. Because partners reported such wide-ranging long-term problems, a psychoeducational support intervention could be tailored to meet their individualized needs.

Second, acceptance and commitment therapy (ACT) is a behavioral intervention that has been used in partners of cancer patients. ACT incorporates mindfulness- and acceptance-based processes to reduce interference of difficult internal experiences (e.g., fear, depressive symptoms) with meaningful activities and QoL that has been used in partners of cancer patients [47]. ACT can help partners clarify and commit to their personal values and be more self-compassionate when experiencing negative thoughts and feelings [48]. Because a large portion of partners in this study reported QoL deficits, this may be an especially beneficial approach to alleviate factors (i.e. depressive symptoms) contributing to those deficits.

Third, meaning-centered psychotherapy has recently been adapted to address the spiritual and existential concerns of cancer caregivers [49]. This approach was originally developed for patients with advanced cancer [50], but it has demonstrated feasibility and acceptability as a web-based psychotherapy for caregivers [51]. Because partners of BCS reported low levels of spiritual behaviors, beliefs, and activities, they may be receptive to an intervention designed to help with existential issues. Using interventions that target partners’ specific problems will be useful in meeting their diverse needs.

## Data Availability

The datasets used and/or analysed during the current study are available from the corresponding author on reasonable request.

## Abbreviations

ACT: Acceptance and Commitment Therapy
BCS: Breast cancer survivors
ECOG-ACRIN: Eastern Cooperative Oncology Group-American College of Radiology Imaging Network
QoL: Quality of life
SPSS: Statistical Package for Social Sciences
